# A Vision-Based Approach for Building Telecare and Telerehabilitation Services

**DOI:** 10.3390/s16101724

**Published:** 2016-10-18

**Authors:** Angela Barriga, José M. Conejero, Juan Hernández, Elena Jurado, Enrique Moguel, Fernando Sánchez-Figueroa

**Affiliations:** Quercus Software Engineering Group, University of Extremadura, Cáceres 10071, Spain; abarrigaj@alumnos.unex.es (A.B.); chemacm@unex.es (J.M.C.); juanher@unex.es (J.H.); enrique@unex.es (E.M.); fernando@unex.es (F.S.-F.)

**Keywords:** fall detection, telerehabilitation, healthcare, 3D-cameras, neural network

## Abstract

In the last few years, telerehabilitation and telecare have become important topics in healthcare since they enable people to remain independent in their own homes by providing person-centered technologies to support the individual. These technologies allows elderly people to be assisted in their home, instead of traveling to a clinic, providing them wellbeing and personalized health care. The literature shows a great number of interesting proposals to address telerehabilitation and telecare scenarios, which may be mainly categorized into two broad groups, namely wearable devices and context-aware systems. However, we believe that these apparently different scenarios may be addressed by a single context-aware approach, concretely a vision-based system that can operate automatically in a non-intrusive way for the elderly, and this is the goal of this paper. We present a general approach based on 3D cameras and neural network algorithms that offers an efficient solution for two different scenarios of telerehabilitation and telecare for elderly people. Our empirical analysis reveals the effectiveness and accuracy of the algorithms presented in our approach and provides more than promising results when the neural network parameters are properly adjusted.

## 1. Introduction

There is no doubt that we are witnessing a major demographic change as elderly people, particularly in developed countries, are increasing in life expectancy. Whereas in the early 20th century, longevity was close to 48 years, recent studies performed in Europe and the U.S. show that the population is increasing its longevity [1,2]. Even more, while in 2013, 18% of Europeans were aged 65 or more (more than 92 million individuals), it is expected that these numbers will considerably grow, being 30% by 2060 [1]. Similar numbers are offered by the counterpart studies of the U.S. government: the population aged 65 and over is projected to be 83.7 million in 2050 [2].

This is why the elderly are assuming a greater importance not only in Europe and the U.S., but also in others countries, like China or Japan. Elderly people are becoming the focus of a wide range of policies and research programs oriented towards strengthening health and social care services for the elderly. Accordingly, the Advisory Group for Health, Demographic Change and Wellbeing of the European Union Framework Programme for Research and Innovation, Horizon 2020, highlights ICT for health to be one of the biggest research challenges to be addressed in the near future. In this setting, gerontechnology is gaining interest as an interdisciplinary field of scientific research in which technology is coping with a wide range of different applications of ICT to provide useful solutions to tackle the needs, aspirations and opportunities for elderly people [3].

Telerehabilitation and telecare are two different scenarios of the application of such technologies, and they refer the idea of enabling people to remain independent in their own homes by providing person-centered technologies to support the individual [4]. Telerehabilitation refers to providing rehabilitation services to people without requiring them to travel to a clinic. On the other hand, telecare denotes remote care of the elderly to allow them to stay in their homes [5]. These technologies share a crucial goal: to assist elderly people at home. Note that they require assistance beyond that habitually needed by an adult because they prefer this kind of care to be provided in their home [6].

Accordingly, the development of such technologies provides wellbeing and health care to the elderly, thus increasing the time they can live at home independently, and this is exactly the focus of this paper. Concretely, this paper focuses on automatic assistance of elderly people at home, and in particular, we evaluate an approach to assist them in their rehabilitation exercises (telerehabilitation) and, additionally, for fall detection (telecare).

### 1.1. Telerehabilitation and Telecare

In the last few years, there have appeared many approaches for telerehabilitation and telecare [7,8,9,10,11,12,13], and according to [14], they may be categorized into two broad groups: wearable devices and context-aware systems. The first category corresponds to kinematic sensor-based systems (KSS) that are somehow worn by the owner on clothing. Because of phones’ built-in sensors, usually accelerometers or gyroscopes, smartphones are included in this category [14,15,16,17]. On the other hand, context-aware systems (CAS) use sensors deployed in the environment to assist people. Video-based systems are included in this category because they use computing vision techniques to gather information from the context in order to detect the person’s movements and react in real time according to those actions [14].

The latest research in the area has shown that the use of body motion sensors that the patient needs to wear for having his/her gestures monitored has proven to be useful research [15,16]. As an example, in [18], Özdemir shows that wearable fall detection systems are extremely efficient, reaching percentages of sensitivity and specificity closer to 100%. However, on the one hand, wearables are not always available for the user; and on the other hand, the use of non-invasive techniques for the elderly provides them a more comfortable scenario. As an example, the study conducted by the University of Utah showed that 80 percent of elderly adults who suffer falls were not able either to prevent or call for help when they had a serious fall, largely because they had not worn the device at the time of falling [19]. By contrast, in context-aware approaches, people do not need to wear any special device to be monitored. Although the performance of these approaches may be lower than KSS ones because they depend on the angle of vision of the camera [14], they can operate automatically, being a non-intrusive approach whilst providing privacy, if the system has the ability to decide whether or not to stream the video [20,21]. Moreover, vision-based systems offer many interesting research applications in this field, and the use of the Kinect-like cameras provides a step forward in telerehabilitation and telecare, allowing patients to be remote and automatically assisted in their particular therapies or attention [7,8,22,23,24,25,26].

### 1.2. Contribution

Accordingly, the use of vision-based approaches provides extensible and versatile solutions to different problems in distinct scenarios, e.g., in the field of gerontechnology (telerehabilitation and telecare), but also in other important ones, such as security (people tracking, video-surveillance, ensuring that emergency exit routes are free and unobstructed by objects, particularly in assisted living), and so on.

In this paper, we present a general approach based on 3D cameras (Kinect-like) and neural network-based algorithms that allows both the detection of static postures for remote and automatically assisting of an elderly person in their rehabilitation exercises in an autonomous way and, additionally, the detection of abnormal behaviors (concretely, fall detections). These two problems may be considered as pattern recognition problems, and this is one of the main novelties of our approach.

The use of 3D cameras allows different points in a body skeleton (head, center shoulder, right/left shoulder, right/left elbows and right/left knees) to be captured by the sensor’s camera. Based on the definition of these points, the flexibility of our approach is illustrated since it may be easily adapted to be used for different problems (or with different purposes) just by changing the points to be captured and adapting the algorithms. Thus, a single camera may replace many wearable sensors (usually not well-accepted by elderly people). The utilization of these points is also crucial to assist elderly people in their rehabilitation exercises or to differentiate normal positions (e.g., sitting) from abnormal behaviors (like falls).

Last, but not least, although being a vision-based solution, the approach presented is non-intrusive, and it respects users privacy, since image recording is not enabled and it does not need human intervention. In that sense, our approach allows the configuration and generation of automatic alarms (like phone calls) when special situations are detected.

The remainder of this paper is organized as follows. Section 2 gives a brief survey on Kinect-based approaches for telerehabilitation and telecare. Section 3 deeply explains the approach we propose to address the aforementioned problems in gerontechnology, namely the detection of static postures for telerehabilitation and fall detection. Section 4 presents the design of the experiments that were performed to evaluate the approach. Section 5 presents the results of the experiments and discusses them. Finally, Section 6 concludes the paper.

## 2. Related Works

The introduction of Kinect cameras has drawn the interests of many researchers to use them in different aspects of gerontechnology. This section focuses on works that try to solve specific problems for telerehabilitation and fall detection through the use of Kinect 3D cameras. An extensive review of other Kinect applications in elderly care and stroke rehabilitation may be found in [27].

In the telerehabilitation area, there are several research papers that have investigated the effects of using Kinect cameras to assist patients with a wide variety of disabilities. Anton et al. [7] developed KiReS (Kinect Rehabilitation System), which uses Kinect as input for both therapists and patients. KiReS uses the facilities of movement recognition and voice commands provided by Kinect for user interaction. Avatars are used to draw the attention to the patient who exercises the proposed movements. Hondori et al. [8] use both inertial and Microsoft Kinect sensors to monitor gestures in stroke patients. They selected eating and drinking as the activities to be monitored because of the special upper body movements that these activities require. Inertial sensors are attached to sensorized utensils (fork, spoon, knife and cup); the Kinect analyzes the movements; and 3D-trajectories are used to determine the success degree of the exercises performed by the patient. The main drawback of these interesting works is that they do not allow remote assistance. Furthermore, they have been designed to solve concrete problems, and they lack the wide scope of a learning machine technique for deep learning.

Similarly, the study performed by Chant et al. [28] shows that game-based applications based on the Kinect are robust tools for spinal cord injury rehabilitation. Their prototype allows measuring and tracking the progress of the patients at home. In [29], a Kinect-based virtual reality training promotes the recovery of upper limb motor function in subacute stroke patients. A recent study [24] was carried out to investigate the effects of using Kinect on stroke survivors with hemiplegia. The results showed a great improvement on upper extremity function compared to the control group that underwent conventional occupational therapy alone. Other studies expanded the use of the Kinect motion sensor system to improve memory performance [25] and functional assessment [26]. Bonnechere et al. reported a validation study [26] of the Kinect sensors in terms of angular measurement comparison with a standard market-based system. They use 3D angles and trajectories to compare both systems, concluding that Kinect sensors may be used instead of market-based systems in planar motions. However, most of these studies relied on the use of games rather than specially-designed scenarios targeting more definite skills. Accordingly, they do not use any deep learning technique for their purposes.

On the other hand, in the fall detection approaches, it is worth mentioning the works of [20,30,31,32]. Mastorakis et al. [31] use the 3D-bounding box of the individual (width, height and depth). Zhang et al. [20] work with the vertical height and the width-height ratio. Finally, Stone et al. [30] and Kwolek at al. [32] also use vertical height, velocity and the accelerometer for fall detection. However, the main drawback of these works is the simplicity in the features they used. Concretely, these features offer enough information for fall detection, but they do not provide sufficient data for solving more general problems like telerehabilitation. Moreover, the methods used to analyze data in these works are decision trees [30,31], SVM [20] and the k-nearest neighbor classifier [32]. From our point of view, the use of a complex neural network, in conjunction with the use of a richer set of features (the coordinates of different body points), makes our work a general approach that can solve different problems in gerontechnology using intelligent depth learning methods.

As a summary, all of these works share the goal of our approach: to take advantage of the potential of 3D cameras in the area of telerehabilitation and fall detection. However, we go a step forward using machine learning techniques that extend the scope of our approach and provide the flexibility for reusing the framework to be applied to other existing problems.

## 3. A Vision-Based Solution for Telecare and Telerehabilitation

Before describing the proposed approach for both, fall detection and autonomous rehabilitation, we make a brief overview for a better understanding of the technologies that we have used in this proposal, namely 3D cameras (time of flight, Kinect-like or depth cameras) and neural networks.

### 3.1. 3D Cameras

Kinect-like cameras have, in addition to a standard camera that provides an RGB image, a depth sensor that allows them to capture a depth map in real time of the scene to survey. Light changes do not affect processing when extracting information from these cameras. Accordingly, segmentation and background subtractions become considerably easier and more accurate once depth information is available. These features allow developers to easily detect and extract human body and movement information. This is the reason why rehabilitation engineers and developers prefer depth cameras over RGB ones in motion capture applications [33].

**Figure 1:**
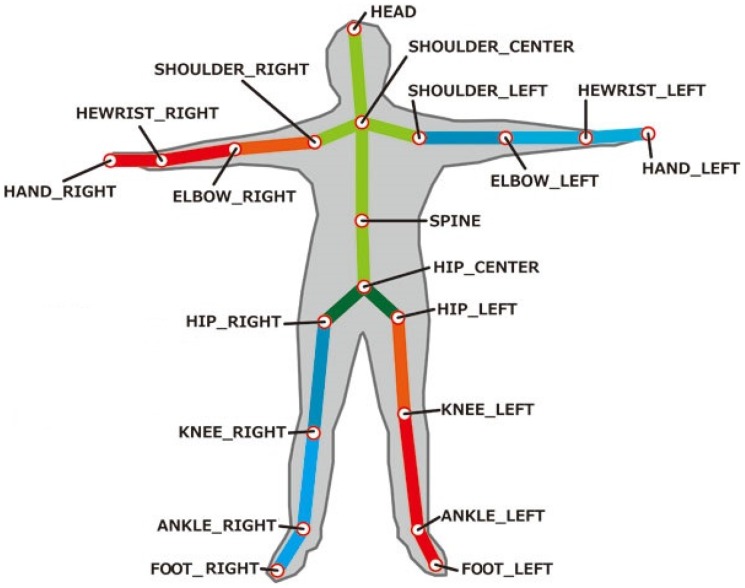
Body joint positions.

In our work, we used the Asus Xtion PRO Live, a Kinect-like camera, which uses OpenNI [34] as the SDK, an open source API that gives developers access to body joint positions and orientations. The Asus Xtion PRO Live sensor can capture RGB, infrared and depth streams with frame rate of 30–60 fps based on resolution. The default display resolution of these streams is 640 × 480 pixels, but it can be increased up to 1280 × 1024 with a lower frame rate. Similarly to Kinect, the Asus Xtion PRO Live depth sensors provide skeleton tracking, which gives developers access to body joint positions and orientations. In Figure 1, the body joint recognition that is provided by OpenNI can be appreciated. OpenNI not only outputs raw images and depth sensor data, but also supports user identification, scene segmentation and skeleton/joint tracking. This last feature is extremely useful for clinicians and therapists to assess the performance of their patients and to track their improvement.

### 3.2. Neural Networks

Artificial neural networks are computational learning models inspired by the central nervous system of human beings. They are generally presented as systems of interconnected artificial neurons, also known as McCulloch–Pitts neurons [35]. An artificial neuron is a mathematical function conceived of as a model of biological neurons. The basic structure of an artificial neuron consists of three different type of units: inputs, outputs and hidden. Input units get information from the environment; outputs send the signal outside the neuron; and hidden are those connections inside the neuron. Interconnected neurons send messages to each other. These connections have numeric weights that change based on the experience of the network, making them adaptive to inputs and capable of learning.

Each neuron gets an amount of inputs and produces an output. This output is created by three functions:

1. A propagation function that generally consists of the sum of each input multiplied by the weight of its connection.
2. An activation function, which modifies the previous function. It is not mandatory, in which case the output would directly be given by the propagation function.
3. A transference function, applied to the value given by the activation function. It is used to limit the output. Some of the most used are sigmoid or hyperbolic tangent functions.

**Figure 2:**
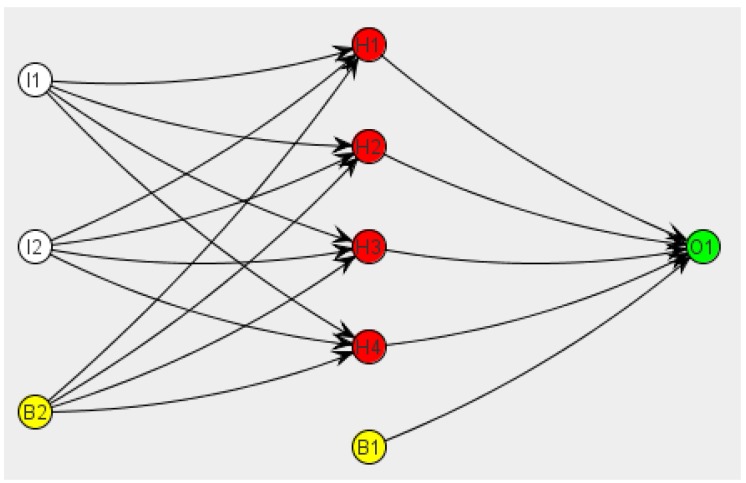
Example of artificial neurons grouped into layers.

Artificial neurons are grouped into layers (Figure 2). Neural networks may be classified by their topology (number and the way layers connect to each other), the learning paradigm (supervised, unsupervised and reinforced learning) and the kind of information they can process (analogical or discrete networks), among others.

A multilayer perceptron is an acyclic multilayer neural network that utilizes a supervised learning technique called backpropagation for training the network. These structures are especially interesting and useful in solving pattern recognition problems [35].

The key concepts of our proposal rely on the consideration of postures detection and fall detection as a pattern recognition problem, a pattern made of a group of coordinates and how they behave together. Since OpenNI is able to capture skeleton and joint coordinates, these data may serve as input to a neural network, expecting to produce as output which posture is being made or if a fall has happened. This is the reason why we decided to use these learning models for our telecare and telerehabilitation vision-based framework, which is shown in the next section.

The mean squared error (Equation (1)) and gradient descent (Equation (2)) are used by the neural network as learning and training functions for the hidden layers. Firstly, the global error of the network is measured by the mean squared error function, the result of which is minimized by the gradient descent function. This function changes the weights of the hidden layers’ connections, making it a backpropagation learning process and forcing the network to recalculate its weights until the desired maximum error is reached.

(1) $$E\left(w_{ij},\theta_{j},w_{kj}^{\prime },\theta_{k}^{\prime }\right)=\frac{1}{2}\sum_{p}\sum_{k}{\left[d_{k}^{p}-f\left(\sum_{j}w_{kj}^{\prime }y_{j}^{p}-\theta_{k}^{\prime }\right)\right]}^{2}$$

(2) $$\begin{array}{cccc} \delta w_{kj}^{\prime } & =-\epsilon \frac{\partial E}{\partial w_{kj}^{\prime }}\\ \delta w_{ji}^{\prime } & =-\epsilon \frac{\partial E}{\partial w_{ji}^{\prime }}\\ \delta w_{kj}^{\prime } & =\epsilon \sum_{p}{△}_{k}^{\prime p}y_{k}^{p} & \mathrm{con} & \;{△}_{k}^{\prime p}=\left[d_{k}^{p}-f\left(v_{k}^{\prime p}\right)\right]\frac{\partial f\left(v_{k}^{\prime p}\right)}{\partial v_{k}^{\prime p}}\\ \delta w_{ij} & =\epsilon \sum_{p}{△}_{j}^{p}x_{i}^{p} & \mathrm{con} & \;{△}_{j}^{p}=\left(\sum_{k}{△}_{k}^{\prime p}w_{kj}^{\prime }\right)\frac{\partial f\left(v_{j}^{p}\right)}{\partial v_{j}^{p}} \end{array}$$

Additionally, we use a sigmoid function (Equation (3)) as the global activation function, which ranges from 0–1. It allows one to normalize all of the values that the network uses, reducing the amount of information and making it computationally efficient.

(3) $$s\left(x\right)=\frac{1}{1+e^{-x}}$$

### 3.3. Static Posture Detection

The purpose of this section is to introduce the different approaches that were taken into account during the development of the proposal. Some of them were discarded during development, but all of them contributed in getting greater knowledge on how to detect static postures, so it is interesting to take a brief look at all of them.

#### 3.3.1. First Approach: Relative Distances between Joints

The idea behind this approach was to get a generic static posture detection based on how relative distances between joints vary when a person alters his or her stance. This way, the detection would work properly with all different types of body shapes.

**Figure 3:**
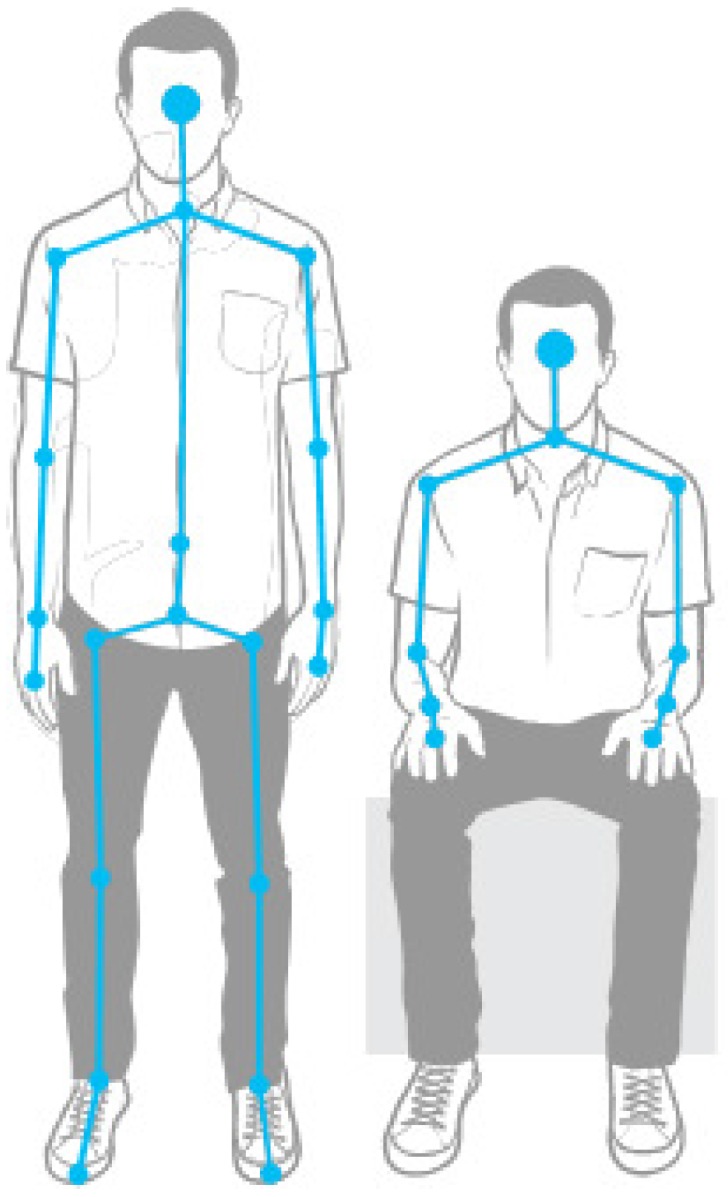
Standing vs. sitting postures.

The work began with the study of the differences between sitting and standing human postures. After analyzing different images of the human body, we concluded that there is a ratio of 2:3 between the head and knees’ distance and the total height for a standing posture, and this proportion is reduced by 50% for a sitting stance (Figure 3).

Then, an experiment was designed in order to check the viability and quality of this approach. The first step was to get the height of the person from the subtraction of the feet and head Y-coordinates. This value needs to be connected with the distance of the user to the camera (Z-coordinate), as the height is relative when this distance varies. After, on each frame, the distance between the head and knees is compared to the threshold of two-thirds of the height; if it is smaller, the person would be sitting and if higher, standing.

The results of this experiment were not good enough to go on with this approach. The system detected properly only 40% of the postures. Body proportions are relative values and offer a low precision because there is much variance between different persons. Moreover use of only a threshold to determinate the solution is extremely simple.

In conclusion, this approach failed because a problem of such magnitude needs more information, more precision and a more complex solution. More joints need to be added to the study, as well as a more potent process capacity.

#### 3.3.2. Second Approach: Neural Networks, Detection of the Whole Body

In order to use a bigger amount of data and a more complex process design, a second approximation to build this framework was developed. A system that included a neural network to work with coordinates of the whole body was designed.

The main goal of this approach is to make the network capable of detecting a posture from the learning extracted from its training. The network is trained with a dataset containing coordinates from the whole body, all labeled according to the posture to which they are related.

After a few tests, this approach showed one fatal problem. The dataset included coordinates from the whole body, even if one joint is not involved in a particular posture. These extra features increase the complexity of the learning process, making the network unable to properly classify different postures. Only the coordinates of the joints involved in a posture must be included in the dataset. For this reason, it was considered a better solution to divide the detection into two parts: upper and lower train. This way, the complexity of the problem is reduced, and only the coordinates that are really needed are used.

#### 3.3.3. Third Approach: Divide and Conquer, Neural Networks with Divided Body Trains

As mentioned above, two programs were designed, from the upper and lower train. Both are similar, varying just in the dataset used for learning and training and the configuration of the neural network (number of inputs, outputs and hidden layers). Algorithm 1 represents a summary of the whole process. This is a generic process that can also be used for detecting abrupt movements, as we will detail in next paragraph. The main difference is the behavior of process *TakeActions* that is detailed in Algorithm 2, for postures detection. In addition, statistical results can be sent remotely to a therapist for further analysis.
**Algorithm 1** Static postures and fall detection algorithm**Require:** CreateDatasetFromTxt(); CreateNeuralNetwork(); TrainNeuralNetwork(); EnableCamera(); **while** (Camera == 0 **and** Person) **do**  ExtractCoordinatesFromCamera();  IntroduceCoordinatesIntoNetwork();  GetOutputFromNetwork();  *TakeActions(); **end**
**while** **Ensure:**
**Algorithm 2** TakeActions algorithm for static postures detection**Require:** SetOfPostures [ ] = GeneratePosturesToBeTrained(); **for** i = 0 **to** sizeOf(SetOfPostures) **do**  count = 0;  **while** (PostureDetected() ≠ SetOfPostures[i]) **and** (count < MaxTrain) **do**   AskUserToTryAgain(SetOfPostures[i]);   count++;  **end**
**while**  **end**
**for** **Ensure:**

As expected, when the dataset size was properly reduced and labeled, the complexity of the learning process decreased, and the results improved dramatically. This way, the upper train program is able to recognize when a person is making the next postures: both arms raised, right arm raised, left arm raised, arms outstretched and both arms down. The joint coordinates used in the dataset come from: both shoulders, elbows and hands, making a total of fifteen coordinates for each postures (and therefore, fifteen input neurons). Based on the detection of these movements and postures, the elderly person may be automatically driven through the rehabilitation exercises.

On the other hand, the lower train program recognizes the main two postures that a person makes with those muscles: standing and sitting. On this occasion, the coordinates taken into account are: both hip sides, knees and foot, making a total of eighteen coordinates.

Finally, and after exhaustive analyzes and tests, it was decided to develop the framework around this approach. It offers a good working solution with low complexity, without losing quality, as we can show in Section 4.

### 3.4. Abrupt Movements

In this section, the design of an abrupt movement detector is introduced, which can be used specifically in fall detection. Although there are differences from the previous static postures detector, the same approach can be used. That is, the work goes on using neural networks and using just joint coordinates involved in the movement, so Algorithm 1 also represents the global performance of the systems.

Moreover, as this detection is more complex, it needs to be divided into two parts. Firstly, posture detection: the system detects whether the user has fallen or not. Secondly, a state machine works in order to discard false alarms and detect only real falls. This way, when the neural network states that it has detected a fall, the state machine analyzes if the individual comes back to a standing position or, otherwise, remains on the ground long enough to send an alarm.

The state machine controls the following stages:

- Normal: When the user is standing, and there are no incidences.
- Abnormal: There has been a fall, but the user has not remained on the ground long enough to activate the alarm. If the user stands, the system goes back to the normal stage.
- Urgent: The user has fallen and remained on the ground for a long time; in a few seconds, an alarm will be activated. If the user stands, the system goes back to the normal stage.
- Emergency: The user has fallen and remained on the ground for enough time to consider it a real emergency. An alarm is activated, and the system cannot go back to normal without manual activation. This way, only an authorized person will be able to turn off the alarm.

In Algorithm 3, a summary of the state machine, i.e., a new version of the *TakeActions* process, is shown.
**Algorithm 3** TakeActions algorithm for fall detection**Require:** **if** (Output == Fall) **then**  StartTimer();  **if** (Timer < SafetyTime **and** Coordinates == StandUpPose) **then**   DiscardAlarm();  **end**
**if**  **if** (Timer > SafetyTime) **then**   UpgradeAlarmLevel();  **end**
**if**  **if** (AlarmLevel == Urgency) **then**   StartAlarm();  **end**
**if** **end**
**if** **Ensure:**

In order to detect falls, the camera extracts coordinates from the head, but the system could be easily adapted to detect any other kind of abrupt movements, such as kicking, punching, running, etc. Only the *TakeActions* process must be adapted (besides training the network, obviously).

### 3.5. System Architecture

This section will present the architecture of the system developed and the flow the programs follow while operating.

As we are talking about a complex system that needs interaction in real time between different components (camera, datasets, neural networks, …) and shares information between them, it was necessary to design an efficient operation flow, both for training neural networks and real-time detection.

Firstly, for training, the camera extracts coordinates of the user to watch over, then these coordinates are normalized by the program and written on an external file to create a dataset.

Secondly, the dataset is given to the neural network for its training. After this process, the system will be ready to work properly.

While operating, during each frame received, the camera will extract the coordinates of the user and introduce them as inputs to the neural network. The neural network will analyze the coordinates provided in each frame and will give an output.

**Figure 4:**
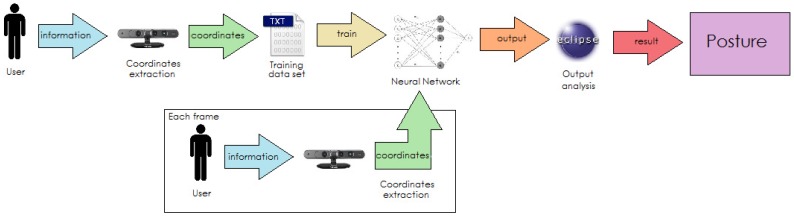
System architecture.

Finally, the program will translate the output of the neural network to a posture, will show it on the screen and act properly according to the specific function of the program (fall detection, helping with rehabilitation, alarm activation, …). This flow process can be noticed in Figure 4.

**Figure A1:**
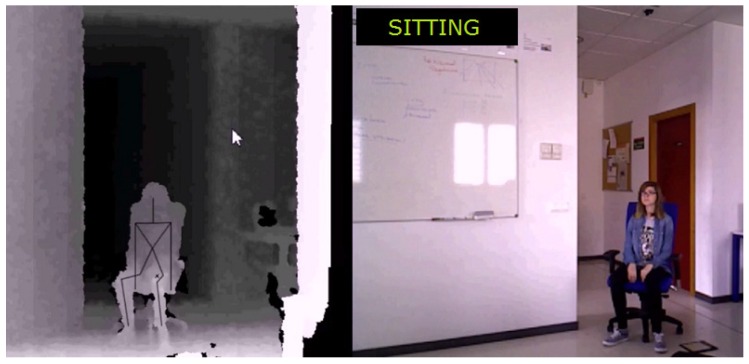
Sitting posture detection.

**Figure A2:**
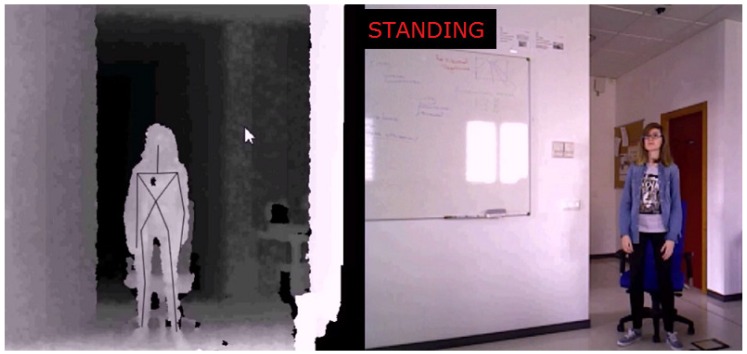
Standing posture detection.

**Figure A3:**
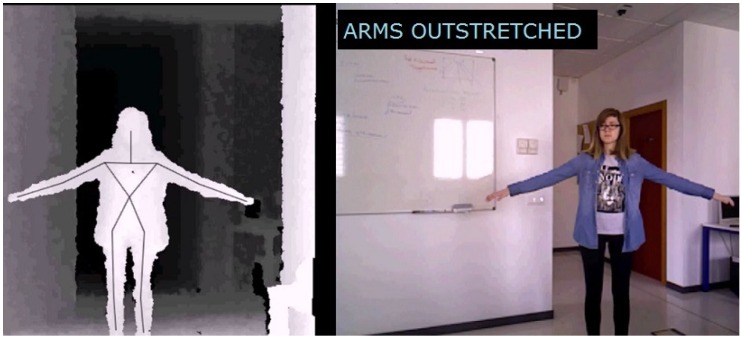
Arm outstretched detection.

**Figure A4:**
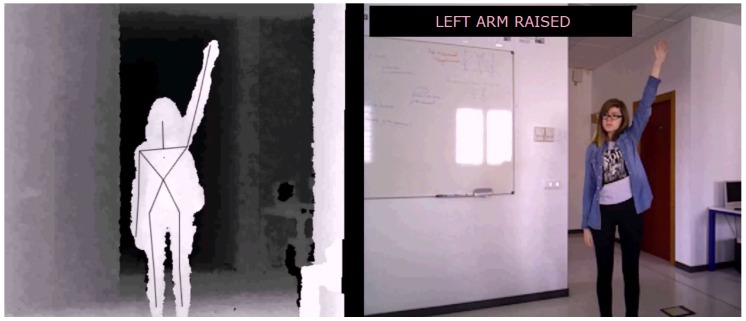
Left arm raised detection.

**Figure B1:**
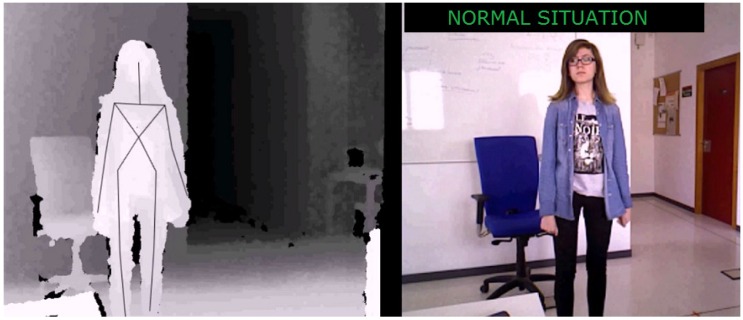
Normal situation detection.

**Figure B2:**
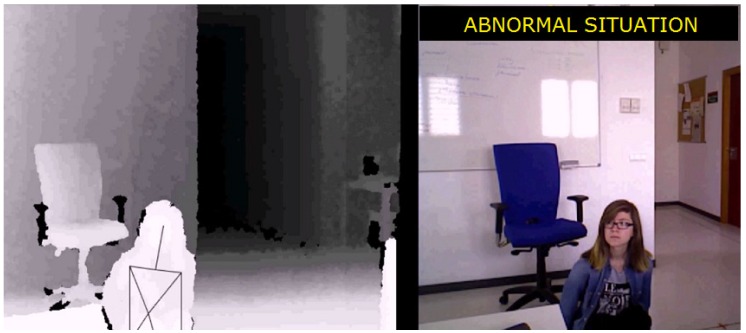
Abnormal situation detection.

**Figure B3:**
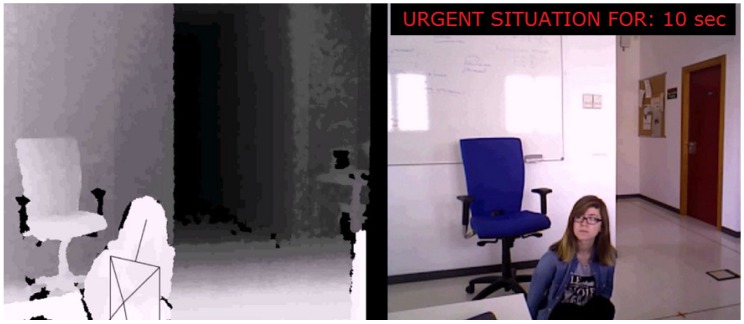
Urgent situation detection.

**Figure B4:**
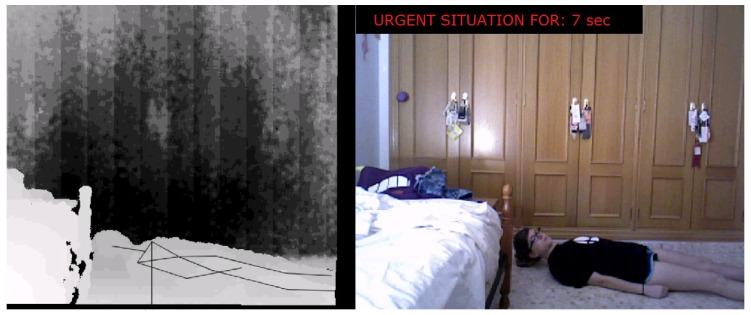
Urgent situation detection.

At the end of this paper, some screenshots of both programs working under our lab conditions are presented (the reader may also find some video examples of the system working at [36]). In Appendix A, Figure A1, Figure A2, Figure A3 and Figure A4 show how the system detects different static postures: sitting, standing, arm outstretched and left arm raised. In Appendix B, Figure B1, Figure B2, Figure B3 and Figure B4 represent a fall detection showing the evolution of the system from a normal to an urgent stage.

## 4. Experimental Design

The main goal of this section is to establish the experiments that will be performed to empirically evaluate the effectiveness and accuracy of the algorithms presented in this approach. The main goal of this evaluation is to perform a study where the algorithms will be tested under different conditions that could make them obtain different results. The study is divided into two different parts, according to the two problems that we try to solve by our approach: (i) the evaluation of the algorithm for the detection of static postures; (ii) the evaluation of the algorithm for fall detection.

Concretely, we formulate the next questions.

Regarding static postures detection:

Q1. What is the best configuration of the neural network learning parameters for the detection of static postures?
Q2. What is the best configuration of the learning set in the neural network for the detection of static postures?

Regarding abnormal behavior detection:

Q3. How does the distance to the camera influence the algorithm for fall detection?
Q4. How does the angle between the camera and the object influence the algorithm for fall detection?

An additional question was also formulated with respect to the characteristics of the subject (person) being detected:

Q5. Do the subject characteristics influence the detection of static positions or abrupt movements?

Based on these questions, four different experiments were developed. In addition, six different subjects with different constitutions, heights and, in general, characteristics participated in the four experiments so that Question Q5 was also evaluated. Likewise, we established a number of recordings, enough to avoid the influence of false positives and negatives in the results obtained (in terms of percentages). Concretely, Table 1 shows a summary of the experiments performed with the subjects involved and the videos obtained. We also analyzed the possibility of using public datasets, where both RGB images and depth maps are available (e.g., [37]), as a benchmark in the evaluation of our algorithms. However, the utilization of these datasets was not ultimately suitable, since they do not provide the information about the coordinates for the different body points.

## 5. Results and Discussion

The goal of this section is two-fold: on the one hand, it shows the main results obtained for each experiment, and on the other hand, these results are discussed for each experiment so that the main conclusions extracted from them are highlighted.

### 5.1. Experiment 1

This experiment was defined to evaluate the influence of the learning parameters of the neural network in the results obtained by the algorithm to detect static postures (Q1). The parameters evaluated are the: number of hidden neurons, maximum error parameter, learning rate and learning function.

#### 5.1.1. Hidden Neurons

Regarding the number of hidden neurons, it is widely claimed in the literature [38] that the optimal structure for a neural network presents the number of hidden neurons that is shown in Equation (4):

(4) Hiddenneurons=((Inputneurons+Outputneurons)/3)*2

Based on this assumption, we defined a set of neural networks with different configurations where the algorithm for the detection of static postures was tested. The algorithm was applied firstly to the upper and, then, to the lower body. Since the results obtained are similar and consistent for the two parts of the body, they are shown in the same table. Table 2 shows these results where the columns describe: (i) the number of hidden neurons, both for upper and lower body (e.g., the first configuration has 12 hidden neurons for the higher body and 10 for the lower one); (ii) the percentage of false positives (situations that should not be identified, but they were); (iii) false negatives (situations that should be identified, but they were not); (iv) true positives (right detections); and (v) true negatives (situations that the algorithm does not detect as static poses and they are really not). The best configuration for the algorithm would be the one with a combination of: (i) the higher value for true positives (and consequently, the lower for false positives); and (ii) the higher value for true negatives (and therefore, the lower for false negatives).

As may be observed in Table 2, in the case where the lower numbers of hidden neurons are selected (Case 1), the neural network learning and detection capabilities are limited. Note, for instance that, in this case, the percentages of true positives and negatives detected are just 35% and 25%, respectively. This is mainly due to the fact that the problem becomes too complex for the computation capability of the neural network. This situation is even more significant when the training set increases its size.

By contrast, in the case with a higher number of hidden neurons (Case 5), the neural network is able to learn, but the results are really difficult to understand. This is mainly due to the training set used (bigger than the one really needed), which makes the results overlapped and many false positives and false negatives appear.

Based on the results, we may observe that the optimal configuration for the neural network corresponds to Case 3 where the best results are obtained. This configuration obtained 95% and 97% of true positives and true negatives, respectively, and the false positives are just 5%. Note that this configuration fulfills the aforementioned requirement about the optimal number of hidden neurons defined in Equation (4).

#### 5.1.2. Maximum Error Parameter

Regarding the maximum error parameter, we also performed several tests with different configurations for this characteristic. In particular, we observed that better results were obtained for tests with lower values for this parameter than those with higher values (obtaining in this case even 80% of errors). However, when tests with lowest values of this parameter are used, the complexity of the network increases exponentially, so that the starting time for the program drastically increases. This problem may be even more important when the threshold is too low, which may cause a program launching fail.

#### 5.1.3. Learning Rate

When the learning rate parameter is too high, the neural weights become too distant, and again, the complexity drastically increases. This situation also implies an increase in learning times and sometimes, even fails in the posture detection (up to 75% of errors). By contrast, a too low learning rate makes the adaptability and learning capabilities of the network decrease, and it may even never learn.

#### 5.1.4. Learning Function

The algorithm for detecting postures has been also tested with two different learning functions: sigmoid and tanh. The tests show that the sigmoid function is the most suitable for the algorithm obtaining a percentage of true positives higher than 80%, whilst the tanh function obtains just 30%.

Concerning subjects characteristics, the tests performed in this experiment did not provide significant differences in the results when different subjects were involved.

### 5.2. Experiment 2

In this experiment, we tested the optimal configuration of the neural network in terms of its learning set. In other words, we evaluated how the group of body coordinates (number of instances in the learning set) influences the results obtained. For instance, when the learning set includes coordinates for joints not related with the postures being detected, the algorithm accuracy significantly decreases (even up to 50% in both the upper and lower body).

Table 3 shows the results obtained in this experiment. The first column presents the number of instances used for the both upper and lower body. These results have been obtained with a neural network with the optimal number of hidden neurons (Case 3 in Experiment 1). Again, due to the similar results obtained for the upper and lower body, they are presented in the same table.

Observe that the configurations with the lowest and highest numbers of instances obtain the worst results. In these cases (1 and 6), the networks do not learn properly because the number of instances is too small or too large, respectively. Table 3 also shows that the optimal learning set is that used in Case 3. In general, when using these networks, a balance in the number of instances must be found with: on the one hand, enough instances for each posture so that the network may differentiate them; on the other hand, not too many instances, since they would add complexity to the learning process, and the results could overlap.

### 5.3. Experiment 3

In this experiment, the algorithm for the fall detection was tested in terms of the distance to the camera. Based on the results obtained in this experiment (together with Experiment 4), an estimation of the cameras needed to be installed in a medium-sized room may be done. Again, in this experiment, different subjects were involved so that the influence of their physical characteristics was also evaluated.

In this case, we evaluated the effectiveness of the algorithm by considering the detection of: (i) true positives (a situation of true fall); and (ii) true negatives (a situation that could be identified as a fall, but is not, like quickly sitting or bending down). These two situations were tested using different distances to the camera and with the camera in a location parallel to the floor. Table 4 summarizes the results obtained. We have categorized the results obtained in only three different cases according to the distances to the camera used in them.

According to the results obtained, it may be observed that the algorithm for fall detections properly works in a distance range between 1 and 4.5 m (Case 2). In most of the situations tested with a distance to the camera lower than 1 m (Case 1), the algorithm obtained low results (10% and 20% of true positives and negatives, respectively), mainly due to the camera not being able to detect the subject’s head. Similarly, when the distance to the camera was higher than 4.5 m (Case 3), the results obtained were even worse than those obtained for Case 1. In this case, although the camera is able to detect the subject, the reduced dimensions identified from these distances make the algorithm dismiss some true falls. Finally, we did not appreciate any difference in the results obtained for the different subjects.

### 5.4. Experiment 4

In this last experiment, the angle between the camera and the subject is evaluated in order to find the best conditions for the detection. In this case, the angle between the camera and the subject was modified in two different directions: modifying the height of the camera with respect to the subject (vertical angle) and making the subject turn over himself or herself (horizontal angle) so that different angles between the camera and the subject’s front side are tested. The results obtained in this experiment are presented in Table 5.

The results obtained in this experiment show how the horizontal angle does not influence the detection of abrupt movements. Note that excellent results are obtained in Cases 1 and 2 where this angle was modified. However, regarding vertical angle, it is clearly evidenced that it highly influences the algorithm. Table 5 shows that the best results are obtained in Case 3, where the camera is placed parallel to the subject or up to $35^{\circ }$ higher. With an angle between $35^{\circ }$ and $45^{\circ }$, the algorithm obtains around 50% of true positives and negatives. When the angle is higher than $45^{\circ }$, the algorithm obtains the worst results (close to 5% of success). Note that in this last case, this angle would imply that the camera is located in a very high position. Similarly, when the camera is situated lower than the subject, the results are acceptable for angles between 0${}^{\circ }$ and $30^{\circ }$ (75% of success). However, when the angle is higher than $30^{\circ }$, the algorithm does not detect the falls with the same accuracy, and the results may not be assured. Again, no differences were detected based on the subjects involved.

### 5.5. Summary

Based on the results obtained in the different experiments, the conclusions that were extracted allow us to answer the research questions formulated in this study:

Q1. What is the best configuration of neural network learning parameters for the detection of static postures?

Based on Experiment 1, we concluded that this algorithm highly depends on the neural networks used. In that sense, the right number of hidden neurons must be selected, and we observed that fulfilling Equation (1) may be a good selection for this number. Regarding the learning parameters, we also concluded that they highly influence the results obtained, and a balanced selection of these parameters must be also selected.

Q2. What is the best configuration of the learning set in the neural network for the detection of static postures?

Based on the results obtained in Experiment 2, we also evidenced the influence of the number of instances in the results obtained. In this case, an intermediate number of instances is the most suitable for the algorithm.

Q3. How does the distance to the camera influence the algorithm for fall detection?

Experiment 3 also provided evidence of how the distance to the camera may influence the results obtained for the fall detection algorithm. For instance, the best configuration for this algorithm is based on a distance to the camera that ranges from 1–4.5 m.

Q4. How does the angle between the camera and the object influence the algorithm for fall detection?

Experiment 4 showed that the horizontal angle does not influence the results obtained by the algorithm. However, vertical angles between the subject and the camera might highly determine those results. In this case, the best results were obtained when the camera was located parallel to the subject.

Q5. Do the subject characteristics influence the detection of static positions or abrupt movements?

Finally, no evidence was observed about the subjects’ influence on the results.

Based on the results obtained in the four experiments, we extracted important conclusions regarding the applicability of the approach. Firstly, we observed that the results obtained by the algorithm for the detection of static postures were successful, at least when the neural network is optimally configured (note that this configuration is managed by the developer). Similarly, the execution environment for this algorithm also influences these successful results, since the user and the camera are located in a controlled position, and thus, their distance and angles do not affect the results. However, regarding the algorithm for fall detection, we observed that the execution environment could jeopardize the success of the results since they are highly influenced by the distance and angles between the camera and the subject. As a summary, while the experiments provided results that confirm the suitability of the approach for the telerehabilitation scenario (static posture detection), the diversity of the execution environments in the telecare scenario (falls detection) may hinder the applicability of the approach to that particular case.

## 6. Conclusions

This paper has presented a general approach for the definition of vision-based pattern recognition solutions. As shown throughout the paper, since the approach is based on the utilization of 3D cameras and neural networks, it benefits from the joined advantages of these two technologies, e.g., distance information and learning capabilities, among others. In this work, the approach has been applied to fall and static posture detection, as special types of telecare and telerehabilitation for elderly people problems, respectively. In that sense, we described how the algorithms built could be used to automatically detect falls and execute alerts or to remotely and automatically assist a patient in his/her daily rehabilitation exercises.

The paper also presented an experimental evaluation where the algorithms were tested under different conditions to check their effectiveness and accuracy. Based on this evaluation, we observed evidence of the correct behavior of the algorithms that provide values close to 100% success when neural network parameters are properly adjusted. We also observed how the camera’s physical location may influence the results obtained and evaluated the suitable distance and angle ranges under the algorithms providing successful results. For instance, we observed that the horizontal angle between the camera and the subject does not affect the results obtained, whilst the vertical angle may highly influence them. In that sense, we concluded that the applicability of the approach for telerehabilitation scenarios could be more promising than in telecare ones. Moreover, the evaluation allowed us to check how the physical characteristics of the subjects detected are not determinant for the algorithm results.

Finally, the flexibility and applicability of the approach has been illustrated by easily adapting the solution for the static postures detection problem to the fall detection one. In that sense, the definition of a general approach also aims at tackling the complexity of coping with further works since solutions to new problems may be provided just by defining different joint points and adapting the algorithms. In particular, we plan to provide new solutions for the elderly domain, but also for new domains, such as security, e.g., providing solutions for automatically detecting dangerous situations in workplaces under special conditions.

## Figures and Tables

**Table 1:** Summary of the experiments performed.

Experiment	Research Questions	Parameter Evaluated	Subjects Recorded	Number of Videos Analyzed
1	Q1 and Q5	Hidden neurons	6	50 (10 for each case in Table 2)
		Maximum error	6	20
		Learning rate	6	20
		Learning function	6	20
2	Q2 and Q5	Instances	6	120 (20 for each case in Table 3)
3	Q3 and Q5	Distance to the camera	6	(30 for each case in Table 4)
4	Q4 and Q5	Angle between camera and subject	6	70 (10 for each case in Table 5)

**Table 2:** Neural networks with different numbers of hidden neurons.

Case	Hidden Neurons (Upper/Lower)	False Positives	False Negatives	True Positives	True Negatives
(1)	8/6	65%	75%	35%	25%
(2)	10/8	20%	5%	80%	95%
(3)	12/10	5%	3%	95%	97%
(4)	14/12	40%	25%	60%	75%
(5)	16/14	90%	85%	10%	15%

**Table 3:** Different number of instances in the learning set.

Case	Instances (Upper/Lower)	False Positives	False Negatives	True Positives	True Negatives
(1)	<300/<400	The network does not learn, since there are not enough instances			
(2)	600–450/700–550	25%	20%	75%	80%
(3)	632/735	5%	3%	95%	97%
(4)	700–900/800–1000	15%	5%	85%	95%
(5)	900–1200/1000–1300	40%	45%	60%	55%
(6)	>1350/>1500	The network does not learn since there are too many instances and it does not discern the pose			

**Table 4:** Distance to the camera in the fall detection algorithm.

Case	Distance to the Camera	True Positives	True Negatives
(1)	<1 m	10%	20%
(2)	1–4.5 m	98%	98%
(3)	> 4.5 m	5%	5%

**Table 5:** Set of angles under the fall detection algorithm tested.

Case	Vertical Angle with Respect to the Subject	Horizontal Angle with Respect to the Subject	True Positives	True Negatives
(1)	$0^{\circ }$	$0^{\circ }$–$45^{\circ }$	98%	99%
(2)	$0^{\circ }$	$45^{\circ }$–$90^{\circ }$	97%	98%
(3)	$0^{\circ }$–$35^{\circ }$ (higher than the subject)	$0^{\circ }$	98%	97%
(4)	$35^{\circ }$–$45^{\circ }$ (higher than the subject)	$0^{\circ }$	50%	50%
(5)	>$45^{\circ }$ (higher than the subject)	$0^{\circ }$	5%	5%
(6)	$0^{\circ }$–$30^{\circ }$(lower than the subject)	$0^{\circ }$	75%	75%
(7)	>30${}^{\circ }$ (lower than the subject)	$0^{\circ }$	5%	5%
